# Epigenetic Silencing of ZNF471 Contributes to Osimertinib Resistance in Lung Adenocarcinoma: β-Hydroxybutyrate Associates with ZNF471 Re-Expression and Reduced Resistance-Associated Phenotypes

**DOI:** 10.32604/or.2026.087712

**Published:** 2026-10-28

**Authors:** Dade Feng, Huiting Yan, Qian Ni, Zirui Peng, Xiong Xu, Yanfang Liang, Chongmei Liu

**Affiliations:** 1 Department of Pathology, Yueyang People’s Hospital of Hunan Normal University, Yueyang, China; 2 School of Basic Medical Sciences, Hunan Normal University Health Science Center, Hunan Normal University, Changsha, China; 3 Department of Orthopedics, Yueyang People’s Hospital of Hunan Normal University, Yueyang, China; 4 Department of Science and Education, Yueyang People’s Hospital of Hunan Normal University, Yueyang, China

**Keywords:** Lung adenocarcinoma, osimertinib resistance, β-hydroxybutyrate, zinc finger protein 471 (ZNF471), DNA methylation, PI3K/AKT/mTOR

## Abstract

**Background:** Acquired resistance limits the benefit of osimertinib in epidermal growth factor receptor (EGFR)-mutant lung adenocarcinoma (LUAD), and epigenetic silencing may contribute to this process. This study aimed to determine whether promoter hypermethylation-associated silencing of zinc finger protein 471 (ZNF471) contributes to resistance and whether β-hydroxybutyrate (BHB) can restore ZNF471 expression. **Methods:** ZNF471 expression and promoter methylation were analyzed in public datasets, clinical samples, and osimertinib-sensitive and resistant cell lines. Functional assays and signaling analyses were performed *in vitro*, and BHB treatment and ZNF471 overexpression were evaluated in xenografts. **Results:** ZNF471 was downregulated and hypermethylated in LUAD, and low expression was associated with advanced stage and poor survival. ZNF471 restoration reduced the osimertinib half-maximal inhibitory concentration, inhibited migration, invasion, and tumorsphere formation, and was associated with suppression of PI3K/AKT/mTOR signaling, whereas ZNF471 knockdown had the opposite effect. BHB increased ZNF471 expression, reduced methylation-specific PCR signals, and synergized with osimertinib in resistant cells *in vitro*. In xenografts, BHB treatment and ZNF471 overexpression each inhibited tumor growth. **Conclusions:** ZNF471 is an epigenetically silenced modulator of osimertinib sensitivity in LUAD. BHB may enhance osimertinib response by reactivating ZNF471. ZNF471 is an epigenetically silenced modulator of osimertinib sensitivity in LUAD. BHB may enhance osimertinib response by reactivating ZNF471. Taken together, these findings suggest that BHB is associated with ZNF471 re-expression and reduced osimertinib resistance-associated phenotypes in LUAD cells, although whether ZNF471 is required for this effect remains to be determined.

## 1 Introduction

Lung cancer remains the leading cause of cancer-related mortality worldwide, with lung adenocarcinoma representing the most common subtype of non-small cell lung cancer [1,2]. Over the past decades, cancer treatment has evolved from surgery and cytotoxic chemotherapy to targeted therapy, immunotherapy, and combination strategies, substantially improving outcomes for many patients [3]. Nevertheless, treatment resistance remains a major challenge. In LUAD, EGFR-targeted therapy, particularly osimertinib, is a standard treatment for patients with activating EGFR mutations, but acquired resistance frequently develops. Activating EGFR mutations define a major molecular subgroup of lung adenocarcinoma and have substantially reshaped its therapeutic landscape through the development of EGFR tyrosine kinase inhibitors. Osimertinib, a third-generation EGFR-TKI, has become a preferred first-line treatment for advanced EGFR-mutant non-small cell lung cancer because of its superior systemic efficacy, central nervous system activity, and survival benefit [1,4,5]. However, acquired resistance inevitably emerges in most patients, severely limiting the long-term clinical benefit of osimertinib [6,7].

Multiple mechanisms have been implicated in osimertinib resistance, including secondary EGFR alterations, MET amplification, bypass pathway activation, epithelial–mesenchymal transition, lineage plasticity, and histological transformation [6,7,8]. Nevertheless, a substantial fraction of resistant tumors lack clearly defined genetic drivers, suggesting that non-genetic and reversible adaptive mechanisms also contribute to therapeutic escape [8,9]. Epigenetic dysregulation is increasingly recognized as a key mediator of cancer cell plasticity, drug-tolerant states, and treatment resistance. In particular, promoter CpG island hypermethylation can transcriptionally silence tumor suppressor genes and thereby promote malignant progression and therapeutic resistance [9,10,11,12]. The PI3K/AKT/mTOR pathway is a central signaling axis downstream of EGFR and other receptor tyrosine kinases in NSCLC. Upon receptor activation, class I phosphoinositide 3-kinase (PI3K) converts phosphatidylinositol-4,5-bisphosphate into phosphatidylinositol-3,4,5-trisphosphate, which recruits and activates AKT through phosphoinositide-dependent kinase 1 and mTOR complex 2. Activated AKT, together with mTOR complex 1 and its downstream effectors, regulates protein synthesis, cell-cycle progression, cellular metabolism, proliferation, and survival, whereas PTEN functions as a major negative regulator of this cascade [13,14]. Genetic alterations and non-genetic activation of this pathway can sustain prosurvival signaling in NSCLC and reduce dependence on EGFR signaling, thereby contributing to primary, adaptive, and acquired resistance to EGFR-TKIs [15]. In particular, reactivation of PI3K/AKT/mTOR signaling has been implicated in resistance to third-generation EGFR-TKIs, including osimertinib [16]. These findings suggest that epigenetically regulated modulators of the PI3K/AKT/mTOR axis may represent important determinants of osimertinib response and resistance. Thus, identifying functionally relevant epigenetically silenced genes may reveal additional mediators of resistance.

Zinc finger proteins are important transcriptional regulators involved in chromatin remodeling, gene expression control, and tumorigenesis [17,18]. As the largest family of transcriptional regulators in mammals, KRAB zinc finger proteins exert context-dependent oncogenic or tumor-suppressive roles across different malignancies through modulation of multiple key signaling pathways including p53, Wnt/β-catenin, and NF-κB [19,20]. ZNF471, a KRAB zinc finger protein, has been reported as a promoter-hypermethylated tumor suppressor in several malignancies. Restoration of ZNF471 suppresses malignant phenotypes and may interfere with oncogenic signaling pathways, including PI3K/AKT/mTOR-related signaling [21,22,23]. However, whether ZNF471 is epigenetically silenced in LUAD and participates in osimertinib resistance remains unexplored.

Because promoter hypermethylation is potentially reversible, interventions that alter methylation-associated chromatin states may restore the expression of silenced genes such as ZNF471. β-Hydroxybutyrate (BHB), an endogenous ketone body, has emerged as a signaling metabolite capable of regulating gene expression through histone deacetylase inhibition, lysine β-hydroxybutyrylation, and DNA methylation-associated mechanisms [24,25,26]. Although BHB has been linked to tumor biology and drug response in a context-dependent manner, its potential role in methylation-associated osimertinib resistance remains unclear.

Given the limited understanding of the epigenetic mechanisms underlying osimertinib resistance in lung adenocarcinoma (LUAD), we aimed to investigate the potential role of ZNF471 in this process. In addition, we sought to explore whether BHB could regulate ZNF471 expression and thereby influence osimertinib sensitivity in LUAD models.

## 2 Materials and Methods

### 2.1 Cell Culture

Human embryonic kidney 293T cells (ATCC, CRL-3216; American Type Culture Collection, Manassas, VA, USA), A549 cells (ATCC, CCL-185), and H1975 cells (ATCC, CRL-5908) were obtained from the American Type Culture Collection. BEAS-2B cells (ATCC, CRL-3588), 16-HBE cells (MilliporeSigma, SCC150, St. Louis, MO, USA), SPCA-1 cells (Procell, CL-0218, Wuhan, Hubei, China), and PC9 cells (ATCC, CRL-32727) were provided by the Cancer Research Institute of Central South University (Changsha, Hunan, China). The osimertinib-resistant derivatives PC9-OR and H1975-OR were provided by the Cancer Research Institute of Central South University. To establish these derivatives, parental PC9 and H1975 cells were exposed to gradually increasing concentrations of osimertinib over an extended period, with intermittent high-dose osimertinib pulses applied during the induction process. The resulting resistant cell populations were designated PC9-OR and H1975-OR, respectively.

All cells were cultured in the appropriate basal medium supplemented with 10% fetal bovine serum (FBS; Procell, 164210, Wuhan, Hubei, China) and 1% penicillin–streptomycin solution (100×; Beyotime, C0222, Shanghai, China). A549 cells were cultured in Dulbecco’s Modified Eagle Medium/Nutrient Mixture F-12 (DMEM/F-12, 1:1; Gibco, C11330500BT, Grand Island, NY, USA). 293T, BEAS-2B, and 16-HBE cells were cultured in Dulbecco’s Modified Eagle Medium (DMEM; Gibco, C11995500BT). H1975, PC9, SPCA-1, PC9-OR, and H1975-OR cells were cultured in Roswell Park Memorial Institute 1640 medium (RPMI 1640; Gibco, C11875500BT). All cells were incubated at 37°C in a humidified atmosphere containing 5% CO_2_. Cell lines were authenticated by short tandem repeat profiling and tested for mycoplasma contamination before use.

### 2.2 Data Acquisition and Preprocessing

RNA-seq gene expression quantification data (STAR-processed TPM) for the TCGA-LUAD cohort were downloaded from the Genomic Data Commons (GDC) Data Portal (https://portal.gdc.cancer.gov; Data Release 45.0). A total of 598 samples, including 539 primary tumor samples and 59 normal samples, were retrieved. Primary tumor cases with complete clinical and survival information (vital status and survival time, defined as days to death or days to last follow-up) were retained for downstream analyses, yielding 522 cases; normal samples were excluded. ZNF471 expression (ENSG00000196263.8) was log2-transformed (value + 1). Differential expression was assessed using the Wilcoxon rank-sum test. Survival analyses were performed in R version 4.2.1 using the survival package (version 3.3.1), survminer package (version 0.4.9), and ggplot2 package (version 3.4.4). The proportional hazards assumption was evaluated using the survival package, and Cox regression was used for survival modeling. The optimal ZNF471 cutoff was determined using the surv_cutpoint function in survminer. DNA methylation β-values generated on the Illumina HumanMethylation450K BeadChip were obtained from UALCAN, and ZNF471 promoter methylation was evaluated using promoter-region probes (e.g., cg00674365). Osimertinib sensitivity data were obtained from the PRISM Repurposing dataset via the DepMap portal (https://depmap.org/portal/; Public 24Q4).

### 2.3 Functional Enrichment Analysis

Functional enrichment analyses were performed using TCGA-LUAD transcriptomic data to identify pathways associated with ZNF471 expression. Primary tumor samples were divided into ZNF471-high and ZNF471-low groups using the same cutoff applied in the survival analysis. Differential expression between the two groups was analyzed using the limma package (version 3.54.2) with an empirical Bayes linear modeling framework, and genes were ranked according to the resulting moderated log2 fold change estimates. Preranked GSEA was performed using GSEA software version 4.4.0 with gene_set permutations (nPerm = 1000) against the Hallmark gene sets from MSigDB release v2026.1.Hs (Broad Institute, https://www.gsea-msigdb.org/gsea/msigdb). The minimum and maximum gene set sizes were set to 15 and 500, respectively. Gene sets with nominal *p* < 0.05 and FDR q < 0.25 were considered significantly enriched, with particular focus on HALLMARK_PI3K_AKT_MTOR_SIGNALING. GSVA was conducted using the GSVA package (version 1.46.0) with default settings for continuous RNA-seq data (method = “gsva”, kcdf = “Gaussian”, mx.diff = TRUE, abs.ranking = FALSE, min.sz = 10, max.sz = 500). GSVA pathway scores were compared between the ZNF471-high and ZNF471-low groups using the Wilcoxon rank-sum test, and *p* values were adjusted using the Benjamini-Hochberg method. Enrichment plots were generated using clusterProfiler (version 4.6.2).

### 2.4 RNA Extraction and Real-Time Quantitative PCR (RT-qPCR)

Total RNA was extracted from PC9, H1975, PC9-OR, and H1975-OR cells using TRIzol reagent (Vazyme, R401-01, Nanjing, Jiangsu, China). For the BHB-treatment experiments, parental PC9 and H1975 cells were treated with 0 or 5 mM β-hydroxybutyrate (BHB) for 24 h, whereas PC9-OR cells were treated with 0, 1, 3, or 5 mM BHB for 24 h before RNA extraction. Unless otherwise indicated, RNA was extracted from untreated PC9, H1975, PC9-OR, and H1975-OR cells. Briefly, cells grown in six-well plates were lysed in 1 mL TRIzol per well, and the lysates were homogenized, phase-separated with chloroform, and centrifuged at 12,000× *g* for 15 min at 4°C. The aqueous phase was collected, RNA was precipitated with isopropanol, washed with 75% ethanol, air-dried, and dissolved in RNase-free water. RNA concentration and purity were assessed using UV spectrophotometry based on the A260/A280 ratio. For reverse transcription, 1 μg of total RNA was used to synthesize cDNA with HiScript II Q RT SuperMix for qPCR (+gDNA wiper) (Vazyme, R223-01) according to the manufacturer’s instructions, and the resulting cDNA was diluted 20-fold with RNase-free water before use. RT-qPCR was performed on a CFX Connect Real-Time PCR System (Bio-Rad, Hercules, CA, USA) using MonAmp ChemoHS qPCR Mix (Monad, MQ00401S, Shanghai, China). Each 20 μL reaction contained 10 μL qPCR mix, 2.5 μL forward primer and 2.5 μL reverse primer (1 μM stock; final concentration, 125 nM each), and 5 μL cDNA. The cycling conditions were 94°C for 30 s, followed by 40 cycles of 94°C for 5 s, 60°C for 15 s, and 72°C for 10 s, with fluorescence acquisition at the end of the extension step. β-actin was used as the internal reference gene because its Ct values were stable across the experimental groups. Relative gene expression was calculated using the 2^−ΔΔCt^ method. Primer sequences are listed in Supplementary Table S1.

### 2.5 Western Blot

Western blotting was performed using protein lysates from BEAS-2B, 16-HBE, SPCA-1, PC9, A549, H1975, PC9-OR, and H1975-OR cells. In addition, PC9-derived vector and ZNF471-overexpressing cells, PC9-OR-derived mock and ZNF471-knockdown cells, including shZNF471#1, shZNF471#2, and shZNF471#3, were analyzed. For β-hydroxybutyrate treatment experiments, PC9-OR cells were treated with increasing concentrations of β-hydroxybutyrate (BHB; 0, 1, 3, or 5 mM) for 48 h or with 5 mM BHB for the indicated time points of 0, 12, 24, 48, and 72 h before protein extraction. Protein concentrations were determined using a bicinchoninic acid (BCA) Protein Assay Kit (Abbkine, KTD3001, Wuhan, Hubei, China). Equal amounts of protein (30 μg per lane) were mixed with 5× protein loading buffer, heated at 95°C for 5 min, and separated by 10% sodium dodecyl sulfate-polyacrylamide gel electrophoresis (SDS-PAGE). The resolved proteins were transferred onto methanol-activated polyvinylidene difluoride (PVDF) membranes at 260 mA for 120 min in an ice bath. The membranes were blocked with 10% non-fat milk in Tris-buffered saline containing 0.1% Tween 20 (TBST) for 2 h at room temperature and incubated overnight at 4°C with the following primary antibodies: rabbit anti-β-actin antibody (Affinity, AF7018, Cincinnati, OH, USA; 1:4000), rabbit anti-ZNF471 antibody (Zenbio, 852105, Chengdu, China; 1:1000), rabbit anti-phospho-PI3K antibody (Affinity, AF3242; 1:1000), mouse anti-PI3K antibody (Proteintech, 60225-1-Ig, Wuhan, China; 1:1000), mouse anti-phospho-AKT antibody (Proteintech, 66444-1-Ig; 1:5000), rabbit anti-AKT antibody (ImmunoWay, YM3618, Suzhou, Jiangsu, China; 1:1000), rabbit anti-phospho-4E-BP1 antibody (Zenbio, R22929; 1:1000), and rabbit anti-4E-BP1 antibody (Zenbio, R24197; 1:1000).

After three washes with TBST, membranes were incubated with HRP-conjugated goat anti-rabbit secondary antibody (Affinity, S0001, 1:6000) or goat anti-mouse secondary antibody (Affinity, S0002, 1:6000) for 1 h at room temperature. Immunoreactive bands were visualized using an enhanced chemiluminescence substrate (Abbkine, BMU102) and captured on a Tanon-5200 chemiluminescence imaging system (Tanon, Shanghai, China). Band intensities were quantified by densitometry using ImageJ software (version 1.54g, NIH, Bethesda, MD, USA) and normalized to β-actin.

### 2.6 Hematoxylin and Eosin (H&E) Staining

Paraffin-embedded tissue sections (4 μm thick) were deparaffinized in xylene (2 × 10 min) and rehydrated through a graded ethanol series of 100%, 90%, 80%, and 70% ethanol, followed by a water rinse. The sections were stained with hematoxylin staining solution (Beyotime, C0107) for 3 min, differentiated for 1 s, and counterstained with eosin staining solution (Beyotime, C0109) for 15 s. After dehydration through 95% ethanol and 100% ethanol, the sections were cleared in xylene and mounted with neutral resin. Images were acquired using a Zeiss Axio Imager.A2 microscope (Carl Zeiss, Oberkochen, Baden-Württemberg, Germany). H&E-stained sections were independently evaluated by a pathologist from Yueyang People’s Hospital who was blinded to the experimental groups. Histopathological assessment was performed according to routine criteria, including tissue architecture, cellular morphology, nuclear atypia, inflammatory infiltration, and necrosis.

### 2.7 Immunohistochemistry

Immunohistochemical (IHC) staining was performed on formalin-fixed, paraffin-embedded tissue sections. The sections were deparaffinized in xylene and rehydrated through a graded ethanol series, followed by rinsing in distilled water. Antigen retrieval was performed by heating the sections in 0.01 M citrate buffer (pH 6.0) at 95°C for 15 min. Endogenous peroxidase activity was blocked by incubating the sections with 3% hydrogen peroxide (H_2_O_2_) in methanol for 30 min at room temperature. After three washes with phosphate-buffered saline (PBS), the sections were blocked with 3% bovine serum albumin (BSA; Solarbio, A8020, Beijing, China) for 1 h at room temperature.

The sections were then incubated overnight at 4°C with the following primary antibodies: rabbit anti-ZNF471 antibody (Zenbio, 852105; dilution, 1:100) and rabbit anti-Ki-67 antibody (Zenbio, R381101; dilution, 1:200). The sections were washed three times with PBS and incubated for 30 min at room temperature with horseradish peroxidase (HRP)-conjugated goat anti-rabbit IgG (H+L) secondary antibody (Proteintech, SA00001-2), diluted 1:500 in antibody diluent. After three additional washes with PBS, immunoreactivity was visualized using a 3,3′-diaminobenzidine (DAB) substrate kit (Proteintech, PR30018).

The sections were counterstained with hematoxylin, dehydrated through a graded ethanol series, cleared in xylene, and mounted. The use of human clinical specimens was approved by the Medical Ethics Committee of Yueyang People’s Hospital (approval no. 2026015). The requirement for written informed consent was formally waived by the committee because this study used archived and de-identified clinical specimens and anonymized clinicopathological information, involved no additional intervention or risk to patients, and did not adversely affect the rights or welfare of the participants. Paired adjacent non-tumor lung tissue sections showing relatively strong ZNF471 immunoreactivity were used as internal positive reference tissues for ZNF471 staining. These tissues served as internal staining references rather than formal external positive controls or independent experimental groups. For the negative staining control, serial sections were processed in parallel using the same staining procedure, except that the primary antibody was omitted and replaced with the corresponding antibody diluent. In the xenograft experiments, the PC9-OR vehicle-treated group served as the biological control for BHB treatment, whereas the PC9-OR empty-vector group served as the vector control for ZNF471 overexpression.

For image selection, five randomly selected fields per section were photographed at 200× magnification, excluding necrotic, folded, detached, or otherwise damaged areas. The fields were selected without reference to staining intensity. Images were captured using a microscope (Olympus, BX53; Tokyo, Japan), and the average optical density of the stained sections was quantified with ImageJ software (version 1.54g, NIH, Bethesda, MD, USA).

### 2.8 Gene Overexpression and Knockdown

The full-length coding sequence of human ZNF471 (NM_020813) was cloned into the PLV3-CMV-MCS-3×FLAG-CopGFP-Puro lentiviral vector (MiaoLingPlasmid, P40122, Wuhan, Hubei, China) to generate the ZNF471-overexpression construct, designated oeZNF471. The empty PLV3-CMV-MCS-3×FLAG-CopGFP-Puro vector was used as the corresponding negative control. For ZNF471 knockdown, short hairpin RNAs targeting ZNF471 were cloned into the pLKO.1-EGFP-Puro lentiviral vector (MiaoLingPlasmid, P1717). The shRNA sequences were as follows: shZNF471#1, 5′-ACATCTTTTTATTCGTCACTGGAGGAGGATTATCATACTGGAGGAGA-3′; shZNF471#2, 5′-CTGCTCAGAAGCGTTTATACAGGAGTATGATGTTGGAGAAG-3′; and shZNF471#3, 5′-AGCCAAACTTCCAATCTTACTCAACATCAAAGAATTCATAC-3′. The empty pLKO.1-EGFP-Puro vector (without shRNA insertion) was used as the mock negative control. Recombinant lentiviruses were produced by cotransfecting 293T cells with the corresponding transfer plasmid, psPAX2 packaging plasmid (MiaoLingPlasmid, P0261), and pMD2.G envelope plasmid (MiaoLingPlasmid, P85101) using Lipo8000™ Transfection Reagent (Beyotime, C0533-1.5mL), according to the manufacturers’ instructions. Virus-containing supernatants were collected 48 h after transfection and used to infect target cells.

For gain-of-function experiments, PC9-OR cells were transduced with lentivirus carrying oeZNF471 or the corresponding empty-vector control. For loss-of-function experiments, PC9 cells were transduced with lentivirus carrying shZNF471#1, shZNF471#2, shZNF471#3, or the empty pLKO.1-EGFP-Puro vector (mock control). Stable transduced cell populations were selected with 2 μg/mL puromycin dihydrochloride (Beyotime, ST551-10mg) for 5 days. The efficiency of ZNF471 overexpression or knockdown was confirmed by RT-qPCR and Western blotting before subsequent experiments.

### 2.9 5-Aza-2′-deoxycytidine Treatment

PC9-OR cells were seeded in six-well plates at a density of 1 × 10^5^ cells/mL and cultured for 24 h. The cells were then treated with 2 μM 5-aza-2′-deoxycytidine (5-Aza; Aladdin, 1476-82-0, Shanghai, China), dissolved in dimethyl sulfoxide (DMSO, Solarbio, D8371) for 96 h. The drug-containing culture medium was replaced every 24 h. At the end of the treatment period, the cells were collected, and genomic DNA was extracted for analysis of the methylation status of the ZNF471 promoter using methylation-specific PCR (MSP).

### 2.10 Colony Formation Assay

Cells in logarithmic growth phase were harvested, resuspended in complete medium consisting of RPMI 1640 medium (Gibco, C11875500BT) supplemented with 10% FBS (Procell, 164210) and 1% penicillin-streptomycin solution (Beyotime, C0222), and counted. PC9-OR cells transduced with oeZNF471 or vector control were seeded into 6-well plates at 2000 cells per well, whereas PC9 cells transduced with shZNF471#1, shZNF471#2, or mock control were seeded at 15,000 cells per well, according to their different clonogenic capacities and to ensure countable colony formation within the assay period. Each well contained 2 mL complete medium, and triplicate wells were prepared for each condition. Plates were gently swirled to distribute the cells evenly and incubated at 37°C in a humidified atmosphere with 5% CO_2_. The medium was replaced every 3 days with fresh complete medium containing osimertinib (TargetMol, 1421373-65-0, Boston, MA, USA; final concentration, 2 μM) or vehicle control (DMSO, Solarbio, D8371). After 14 days, the cells were washed twice with phosphate-buffered saline (PBS), fixed with methanol for 15 min at room temperature, washed again with PBS, and stained with 0.1% crystal violet solution (Biosharp, BL802A, Hefei, Anhui, China) for 20 min. Excess stain was removed by rinsing with PBS, and the plates were air-dried and photographed using an upright microscope (Zeiss Axio Vert.A1, Carl Zeiss, Oberkochen, Baden-Württemberg, Germany).

### 2.11 Tumorsphere Formation Assay

Single-cell suspensions of PC9-OR/vector, PC9-OR/oeZNF471, PC9/mock, PC9/shZNF471#1, and PC9/shZNF471#2 cells were seeded in ultra-low-attachment 12-well plates at a density of 2000 cells per well in 2 mL serum-free DMEM (Gibco, C11995500BT) supplemented with 1× B27 supplement without vitamin A (Thermo Fisher Scientific, 12587010, Carlsbad, CA, USA), recombinant human EGF (Abbkine, PRP100159), and recombinant human bFGF (Abbkine, PRP1010), with no FBS added (0%). The cells were cultured at 37°C in a humidified incubator containing 5% CO_2_ for 10 days. Tumorspheres with a diameter >50 μm were counted manually under a light microscope (Olympus, BX53) in the entire well rather than in predefined microscopic fields. Each condition was analyzed in three technical replicate wells and repeated in three independent biological experiments.

### 2.12 Cell Counting Kit-8 (CCK-8) Assay and Combination Index (CI)

PC9-OR/vector, PC9-OR/oeZNF471, PC9/mock, PC9/shZNF471#1, PC9/shZNF471#2, PC9-OR, and H1975-OR cells were seeded in 96-well plates and allowed to attach overnight. For the osimertinib dose-response assay, cells were seeded at 2000 cells per well and treated for 48 h with serial dilutions of osimertinib (10, 40, 60, 100, 1000, 10,000, 20,000, 60,000, 100,000, and 200,000 nM). Cell viability was assessed using Cell Counting Kit-8 (CCK-8) (Tsbiochem, C0005, Shanghai, China), and 10 μL of reagent was added to each well followed by incubation at 37°C for 2 h. Absorbance at 450 nm was measured using a Synergy 2 microplate reader (BioTek, Winooski, VT, USA). IC50 values were calculated by nonlinear regression using GraphPad Prism 9.5 (GraphPad Software, San Diego, CA, USA).

For combination studies, PC9-OR and H1975-OR cells were seeded at 1500 cells per well and treated for 48 h with BHB sodium salt (Aladdin, H121822), osimertinib at a fixed concentration of 4 μM, or their combination. DMSO served as the vehicle control for osimertinib, and matched solvent controls were included for BHB. Drug synergy was evaluated using CompuSyn software (version 1.0; ComboSyn, Inc., Paramus, NJ, USA) based on the Chou-Talalay median-effect principle. Each condition was tested in triplicate wells and repeated in three independent biological experiments [27].

### 2.13 Transwell Assay

Cell migration and invasion were assessed using Transwell cell culture inserts (24-well format, polycarbonate membrane, 6.5-mm diameter, 8.0-μm pore size; LABSELECT, 14301, Beijing, China). For the migration assay, cells were serum-starved for 24 h, harvested, and resuspended in serum-free medium at a density of 2.5 × 10^5^ cells/mL. A 200-μL cell suspension was added to the upper chamber, and 500 μL complete medium containing 10% FBS was added to the lower chamber as a chemoattractant. Cells were incubated at 37°C for 24 h.

For the invasion assay, Matrigel (ABW, 082706, Shanghai, China) was thawed at 4°C, diluted 1:8 in prechilled serum-free medium, and used to coat the upper surface of each insert. After polymerization at 37°C for 1–3 h, 200 μL of the cell suspension (2.5 × 10^5^ cells/mL in serum-free medium) was added to the upper chamber, and 600 μL complete medium containing 10% FBS was added to the lower chamber. Cells were incubated at 37°C for 48 h. After incubation, non-migrated or non-invaded cells on the upper surface of the membrane were removed with cotton swabs. Cells on the lower surface were fixed with methanol for 15 min and stained with 0.1% crystal violet for 10 min. The membranes were then washed three times with PBS, air-dried, and photographed under a microscope (Olympus, BX53). Three random fields per insert were captured for quantification. Image analysis was performed using ImageJ (version 1.54g, NIH, Bethesda, MD, USA). Each condition was tested in triplicate inserts and repeated in three independent biological experiments.

### 2.14 RNA Sequencing (RNA-Seq)

A549 and SPCA-1 cells were treated with 5 mM BHB sodium salt (Aladdin, H121822) or an equal volume of saline vehicle for 48 h. After treatment, the cells were washed once with ice-cold phosphate-buffered saline (PBS), and total RNA was extracted using TRIzol reagent (Vazyme, R401-01) according to the manufacturer’s instructions.

RNA-seq library preparation and sequencing were performed by Shanghai Majorbio Bio-pharm Technology Co., Ltd. (Shanghai, China). Sequencing libraries were prepared using the TruSeq RNA Sample Preparation Kit (Illumina, RS-301-2002, San Diego, CA, USA). Double-stranded cDNA was synthesized using the SuperScript double-stranded cDNA synthesis kit (Invitrogen, 11917010, Carlsbad, CA, USA), and the libraries were amplified using Phusion DNA polymerase (New England Biolabs, M0535, Ipswich, MA, USA). The libraries were subsequently sequenced on an Illumina NovaSeq 6000 platform (Illumina, San Diego, CA, USA).

Raw sequencing reads were processed using SeqPrep (v1.2) and Sickle (v1.33) to remove adaptor sequences and low-quality reads. The resulting clean reads were aligned to the human reference genome hg38 using HISAT2 (v2.2.1), with gene annotation files obtained from Ensembl. The aligned reads from each sample were assembled and quantified using StringTie (v3.0) in a reference-guided manner. Transcript abundance was expressed as transcripts per million (TPM). Differential expression analysis was performed using the edgeR (v4.10.5) package in R (v4.4.2), with *p* values adjusted using the Benjamini–Hochberg false discovery rate procedure. Genes with |log_2_ fold change| > 0.58 and adjusted *p* < 0.05 were considered differentially expressed. Three independent biological replicates were analyzed for each cell line and treatment group. The RNA-seq data have not yet been deposited in a public repository and will be made available upon publication.

### 2.15 Proteomic Analysis

Quantitative proteomic analysis was performed by PTM Biolabs/Jingjie Bio using a standard LC–MS/MS workflow. H1975 and A549 cells were treated with 5 mM BHB sodium salt or an equal volume of saline vehicle for 24 h. Cell pellets were lysed in 8 M urea containing 1% protease inhibitor cocktail, sonicated on ice with a high-intensity ultrasonic processor (Scientz, Ningbo, Zhejiang, China), and clarified by centrifugation at 12,000× *g* for 10 min at 4°C. Protein concentrations were determined by BCA assay. Proteins were precipitated with 20% trichloroacetic acid, washed three times with precooled acetone, redissolved in 200 mM TEAB, reduced with 5 mM dithiothreitol for 30 min at 56°C, alkylated with 11 mM iodoacetamide for 15 min at room temperature in the dark, and digested overnight with trypsin at a 1:50 enzyme-to-protein ratio. The resulting peptides were desalted using Strata X SPE columns and analyzed on a NanoElute UHPLC system coupled to a timsTOF Pro 2 mass spectrometer (Bruker Daltonics, Bremen, Germany) operating in dia-PASEF mode. Peptides were separated on a home-made reversed-phase analytical column (25 cm × 100 μm i.d.) using solvent A (0.1% formic acid, 2% acetonitrile in water) and solvent B (0.1% formic acid in acetonitrile) with a gradient of 6%–24% B for 0–14 min, 24%–35% B for 14–16 min, 35%–80% B for 16–18 min, and 80% B for 18–20 min at 500 nL/min. The electrospray voltage was 1.75 kV. Raw DIA data were processed using Spectronaut v18 against the Homo_sapiens_9606_SP_20231220.fasta database (20,429 entries). Trypsin/P was specified as the cleavage enzyme with up to two missed cleavages. Carbamidomethylation of cysteine was set as a fixed modification, and N-terminal acetylation and methionine oxidation were set as variable modifications. Protein, peptide, and PSM false discovery rates were controlled at <1%. No TMT labeling, HPLC fractionation, or affinity enrichment was performed.

### 2.16 Bisulfite Modification and Methylation-Specific PCR (MSP)

Genomic DNA was extracted using the Blood/Cell/Tissue Genomic DNA Extraction Kit (ELK Biotechnology, EP007, Wuhan, China). For bisulfite conversion, 500 pg to 2 μg genomic DNA in a 20 μL volume was processed with the EZ DNA Methylation-Gold Kit (Zymo Research, D5005, Irvine, CA, USA) according to the manufacturer’s protocol. The converted DNA was eluted in 20 μL M-Elution Buffer, and 2 μL was used as the template for MSP. Each 20 μL PCR reaction contained 10 μL 2× HieffTM PCR Master Mix (With Dye) (Yeasen, 10102ES08, Shanghai, China), 1 μL each of forward and reverse primers (10 μM), 2 μL bisulfite-converted DNA, and 6 μL nuclease-free water. Amplification was performed under the following conditions: 98°C for 4 min; 40 cycles of 98°C for 30 s, 56°C for 30 s, and 72°C for 30 s; and a final extension at 72°C for 10 min. Fully methylated and unmethylated DNA controls were included as positive and negative controls, respectively, and a no-template control was used to monitor contamination. PCR products were separated on 2% agarose gels and visualized under UV light. Methylation status was assessed qualitatively based on the presence or absence of the expected methylated and unmethylated bands; densitometric quantification was not performed. Primer sequences are listed in Supplementary Table S1.

### 2.17 Wound Healing Assay

PC9-OR cells were seeded in 6-well plates at a density of 5 × 10^5^ cells/well and cultured until 90–100% confluence. A uniform scratch was created using a sterile 200 μL pipette tip, and detached cells were removed by washing twice with PBS. Cells were then incubated in serum-free medium containing 0, 1, 3, or 5 mM BHB sodium salt. To minimize the contribution of cell proliferation to wound closure, the assay was performed in serum-free medium and limited to a 24 h observation period; no additional proliferation inhibitor was used. Phase-contrast images were captured at 0 and 24 h from the same fields using an inverted microscope (Olympus IX73, Olympus, Tokyo, Japan). Wound area was quantified using ImageJ (version 1.54g, National Institutes of Health, Bethesda, MD, USA), and wound closure was calculated as [(area_0h − area_24h)/area_0h] × 100%. Each condition was analyzed in three independent biological replicates, with three technical replicate wells per condition.

### 2.18 Animal Models

Forty female BALB/c nude mice (4 weeks old and weighing approximately 18–19 g) were obtained from Silaike Jingda Laboratory Animal Co., Ltd. (Changsha, Hunan, China). The mice were housed under specific pathogen-free conditions at 22 ± 2°C and 50–60% relative humidity under a 12-h light/dark cycle. They had free access to standard rodent chow and sterilized water. Each mouse was considered one experimental unit. The mice were randomly assigned to four groups, with 10 mice per group.

Parental PC9-OR cells, PC9-OR cells stably transduced with the empty vector (PC9-OR-vector), or PC9-OR cells stably overexpressing ZNF471 (PC9-OR-oeZNF471) were suspended in phosphate-buffered saline (PBS) and subcutaneously inoculated into the right flank at a dose of 2 × 10^6^ cells in 100 μL per mouse. The four experimental groups were defined as follows: (i) the parental PC9-OR + vehicle group, in which mice were inoculated with parental PC9-OR cells and received PBS by intraperitoneal injection; (ii) the parental PC9-OR + β-hydroxybutyrate (BHB) group, in which mice were inoculated with parental PC9-OR cells and received BHB sodium salt (Aladdin, H121822) at 100 mg/kg by intraperitoneal injection; (iii) the PC9-OR-vector group, in which mice were inoculated with PC9-OR-vector cells and received no intraperitoneal PBS or BHB treatment; and (iv) the PC9-OR-oeZNF471 group, in which mice were inoculated with PC9-OR-oeZNF471 cells and received no intraperitoneal PBS or BHB treatment.

BHB treatment was initiated on day 10 after tumor-cell inoculation and continued once daily through day 35. Mice in the parental PC9-OR + vehicle group received PBS by intraperitoneal injection according to the same schedule. Tumor dimensions, including length and width, and body weight were measured every 5 days beginning on day 10 after tumor-cell inoculation by an investigator who was blinded to group allocation. Tumor volume was calculated using the following formula: tumor volume = 0.5 × width^2^ × length. Tumor volume, tumor weight, and body weight were prespecified outcome measures. On day 35 after tumor-cell inoculation, or earlier if a humane endpoint was reached, the mice were euthanized by an overdose of sodium pentobarbital. The tumors were then excised, weighed, and photographed. Tumor tissues were fixed in 10% neutral buffered formalin, embedded in paraffin, and sectioned for subsequent immunohistochemical analysis.

A predefined humane endpoint was established as a tumor volume of 2000 mm^3^. Any mouse whose tumor volume reached or exceeded 2000 mm^3^ was euthanized immediately. For immunohistochemical analysis, the slides were coded before evaluation. The staining intensity and percentage of positive cells were independently assessed by two investigators who were unaware of the experimental group assignments. All animal experiments, including the nude mouse xenograft assays, were approved by the Biomedical Research Ethics Committee of Hunan Normal University (approval no. D2025046) and were conducted in accordance with the approved institutional protocol, the ARRIVE guidelines, and institutional guidelines for the care and use of laboratory animals.

### 2.19 Statistical Analysis

Statistical analysis was performed using GraphPad Prism 9.5 (GraphPad Software). Data are presented as mean ± standard deviation (SD). Comparisons between two groups were evaluated with an independent-samples *t*-test or the Mann–Whitney U test, depending on data distribution. Differences among multiple groups were assessed by one-way analysis of variance (ANOVA) followed by Tukey’s *post-hoc* test. A two-sided *p* < 0.05 was considered statistically significant.

## 3 Results

### 3.1 ZNF471 Is Downregulated in Lung Adenocarcinoma and Is Associated with Favorable Clinical Outcome

Bioinformatics analysis of the TCGA-LUAD dataset revealed that ZNF471 was significantly downregulated in LUAD tissues compared to normal controls (Fig. 1A), a finding further validated in paired tumor-adjacent specimens (Fig. 1B). Immunohistochemical staining and quantitative analysis of self-collected clinical specimens confirmed reduced ZNF471 protein expression in LUAD tissues (Fig. 1C). Consistently, Western blot analysis indicated lower ZNF471 protein expression in LUAD cell lines than in normal bronchial epithelial cells (Fig. 1D). Furthermore, ZNF471 expression was negatively correlated with pathologic stage, decreasing progressively with tumor advancement (Fig. 1E). Survival analysis revealed that patients with high ZNF471 expression exhibited significantly better overall survival (OS; HR = 0.68, 95% CI: 0.49–0.95, *p* = 0.024) and disease-specific survival (DSS; HR = 0.62, 95% CI: 0.43–0.91, *p* = 0.015) than those with low expression (Fig. 1F,G). Collectively, these findings indicate that ZNF471 downregulation is closely associated with unfavorable prognosis in LUAD.

**Figure 1:**
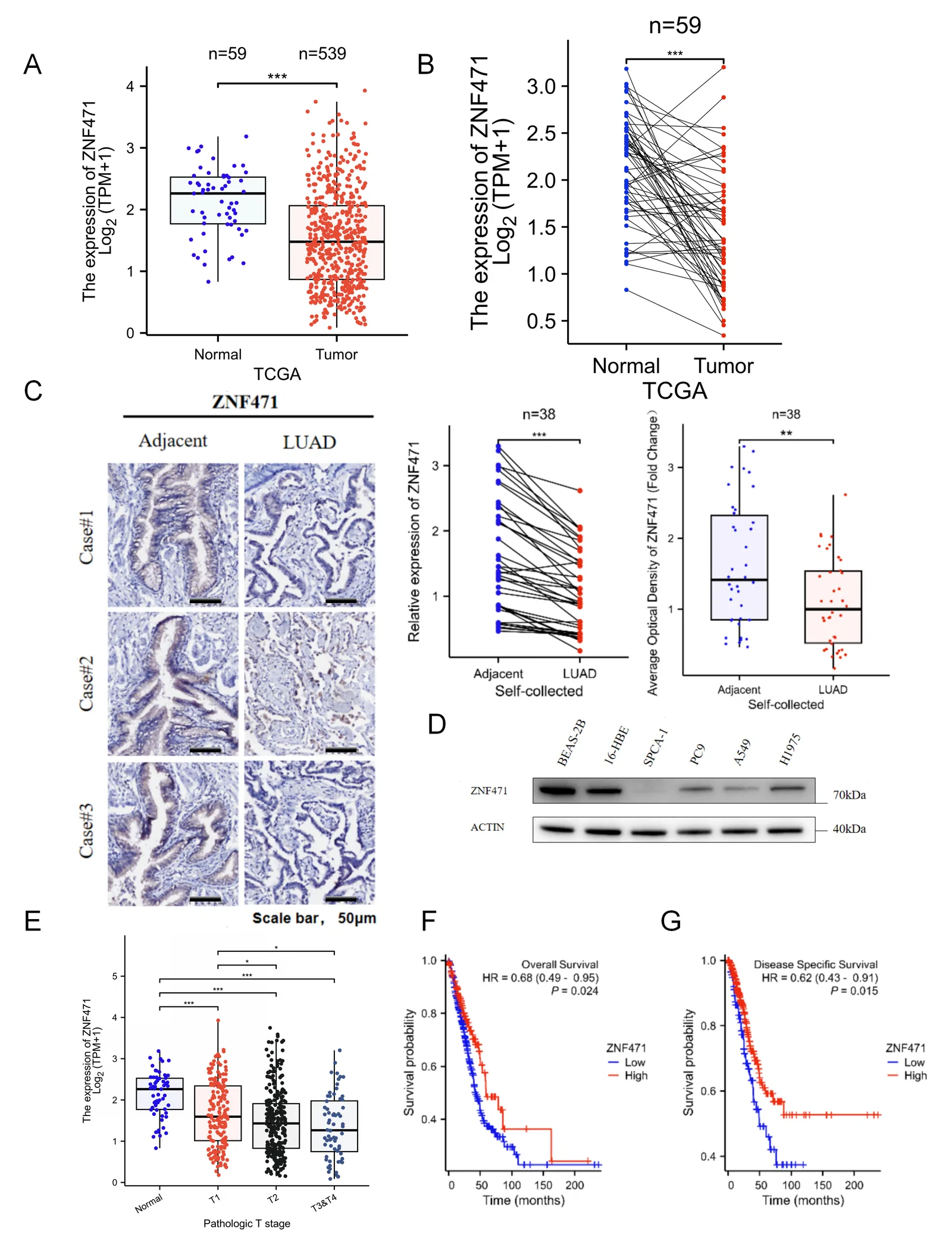
ZNF471 is downregulated in lung adenocarcinoma (LUAD) and associated with prognosis. (**A**) ZNF471 mRNA expression levels in normal lung tissues (n = 59) and LUAD tissues (n = 539) from the cancer genome atlas (TCGA) database. (**B**) ZNF471 mRNA expression in paired adjacent non-tumor and tumor tissues from TCGA-LUAD cohort (n = 59). (**C**) Representative IHC images and AOD quantification of ZNF471 in paired adjacent non-tumor and LUAD tissues from self-collected samples. (**D**) Western blot analysis of ZNF471 protein levels in normal human bronchial epithelial cell lines (BEAS-2B and 16-HBE) and LUAD cell lines (SPCA-1, PC9, A549, and H1975). β-actin served as the loading control. (**E**) ZNF471 mRNA expression across pathologic stages in TCGA-LUAD. (**F**) Kaplan–Meier survival curves showing overall survival (OS) of LUAD patients in the TCGA cohort stratified by ZNF471 mRNA expression levels (low vs. high) using the optimal cutoff value determined by the minimum *p*-value approach. (**G**) Disease-specific survival analysis stratified by ZNF471 expression. Statistical significance is denoted by *p < 0.05, **p < 0.01, ***p < 0.001.

### 3.2 ZNF471 Promoter Hypermethylation Is Associated with Transcriptional Repression in Lung Adenocarcinoma

Having established that ZNF471 is significantly downregulated in LUAD, we next sought to elucidate the epigenetic mechanism underlying its transcriptional silencing.

Analysis of seven specific CpG sites within the promoter CpG island revealed significantly higher methylation in tumor tissues than in paired normal controls (Fig. 2A). TCGA-LUAD data showed higher ZNF471 promoter methylation in primary tumors than in normal lung tissues (Fig. 2B). At the cellular level, methylation-specific PCR revealed predominantly unmethylated bands in 16-HBE cells, whereas SPCA-1, A549, and PC9 cells showed methylated patterns (Fig. 2C). Collectively, these findings suggest that promoter CpG island hypermethylation contributes to ZNF471 transcriptional repression in LUAD.

**Figure 2:**
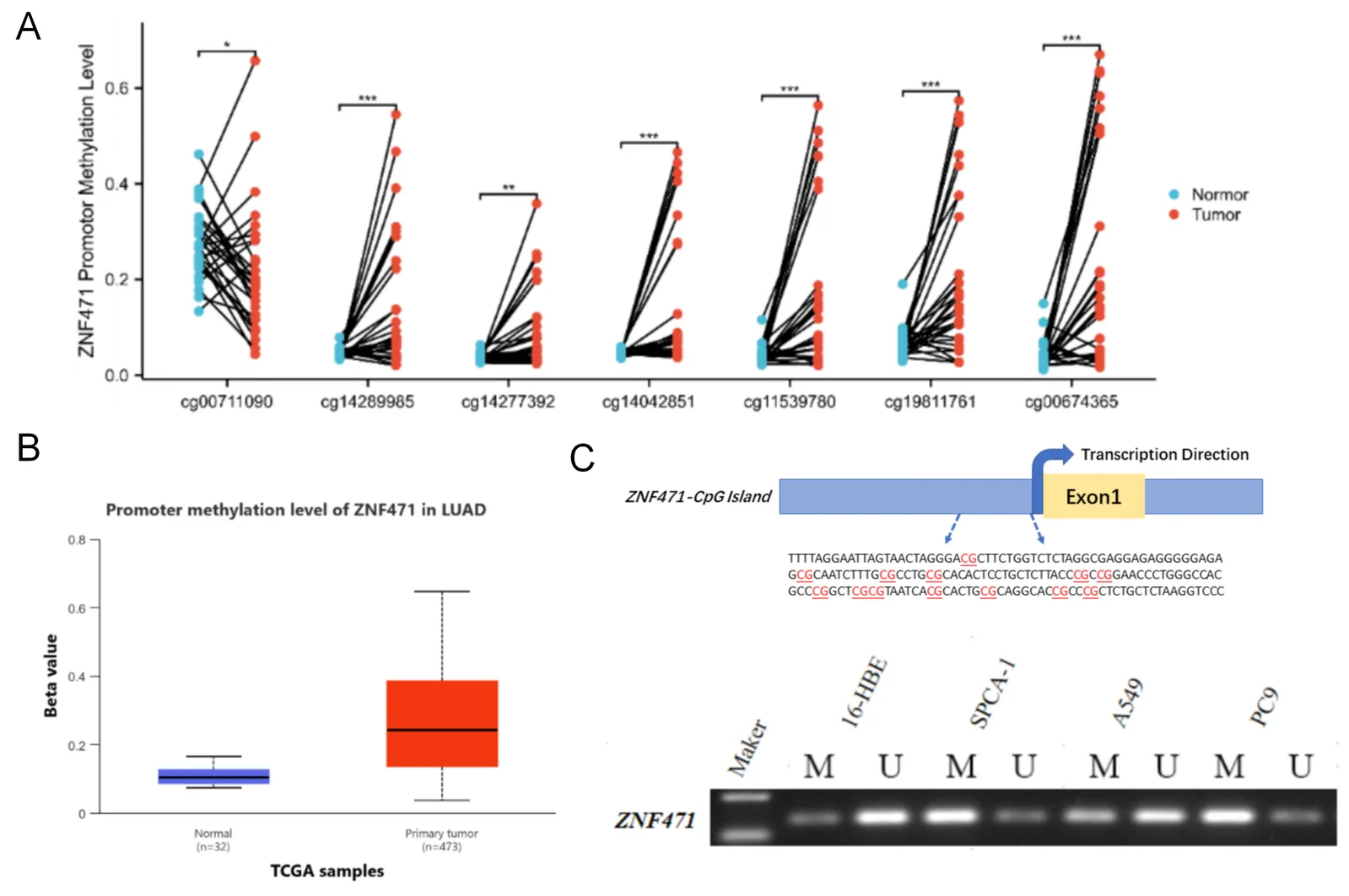
ZNF471 promoter hypermethylation is associated with reduced ZNF471 expression in lung adenocarcinoma (LUAD). (**A**) Methylation levels of seven CpG sites (cg00711090, cg14289985, cg14277392, cg14042851, cg11539780, cg19811761, and cg00674365) in paired normal and tumor tissues. (**B**) Promoter methylation level (Beta value) of ZNF471 in normal lung tissues (n = 32) and LUAD primary tumors (n = 473) from TCGA database. (**C**) Top: schematic diagram of the ZNF471 promoter CpG island, showing the transcription direction, exon 1 (Exon1), and CpG site distribution. Bottom: Methylation-specific PCR (MSP) analysis of ZNF471 promoter methylation status in normal bronchial epithelial cell line (16-HBE) and LUAD cell lines (SPCA-1, A549, and PC9). M: methylated band; U: unmethylated band. Statistical significance is denoted by *p < 0.05, **p < 0.01, ***p < 0.001.

### 3.3 ZNF471 Overexpression Reverses Osimertinib Resistance and Attenuates Malignant Phenotypes in Lung Adenocarcinoma

Having established that ZNF471 is epigenetically silenced via promoter hypermethylation in LUAD, we next investigated whether this downregulation functionally contributes to osimertinib resistance. To this end, ZNF471 was overexpressed using an expression vector, whereas knockdown was achieved using three independent shRNAs targeting ZNF471 (shZNF471-1, shZNF471-2, and shZNF471-3; Supplementary Table S2); the corresponding empty vector and scrambled shRNA (shNC) were used as negative controls. The analysis revealed that, compared to patients with stable disease (SD), those with progressive disease (PD) under therapy exhibited significantly lower ZNF471 expression (Fig. 3A, left). Moreover, Spearman correlation analysis demonstrated a negative correlation between ZNF471 expression and osimertinib IC_50_ values across LUAD cell lines (Fig. 3A, right), suggesting that reduced ZNF471 is associated with impaired drug sensitivity.

Upon examining parental and osimertinib-resistant LUAD cell lines, we found that ZNF471 protein and mRNA levels were markedly downregulated in resistant derivatives compared with their parental counterparts (Fig. 3B). To functionally validate its role, we restored ZNF471 expression in resistant cells and depleted it in sensitive cells. ZNF471 overexpression significantly decreased the osimertinib IC_50_ in resistant cells, whereas its knockdown substantially increased the IC_50_ in sensitive cells (Fig. 3C,D).

Consistently, ZNF471 restoration suppressed colony formation, sphere formation, and migratory and invasive capacities in resistant cells under osimertinib treatment, while its depletion exerted opposite effects in sensitive cells (Fig. 3E–G). Quantitative analyses in Supplementary Fig. S1 further confirmed that ZNF471 overexpression reduced colony formation, decreased mean tumorsphere diameter, and inhibited Transwell migration and invasion, while ZNF471 knockdown produced reciprocal effects. Collectively, these reciprocal gain- and loss-of-function findings support a functional role for ZNF471 in restraining osimertinib resistance-associated phenotypes in LUAD cells.

**Figure 3:**
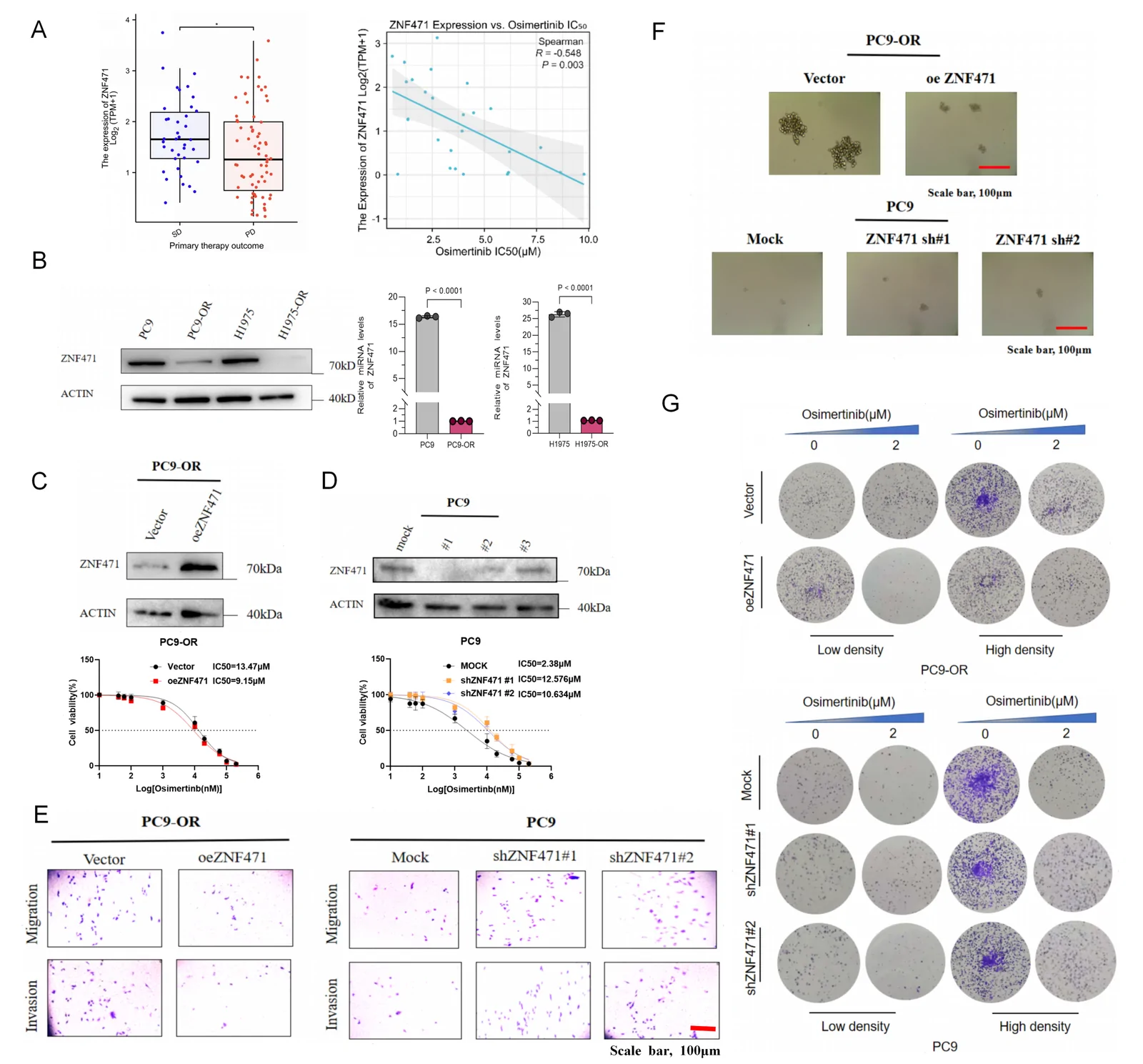
ZNF471 restoration attenuates osimertinib resistance-associated phenotypes in lung adenocarcinoma (LUAD) cells. (**A**) Left: ZNF471 mRNA expression in LUAD patients with stable disease (SD) versus progressive disease (PD) under osimertinib therapy. Right: Spearman correlation analysis between ZNF471 expression and osimertinib half-maximal inhibitory concentration (IC_50_) in LUAD cell lines. (**B**) Left: western blot analysis of ZNF471 protein levels in parental (PC9 and H1975) and osimertinib-resistant (PC9-OR and H1975-OR) LUAD cell lines. Right: quantitative polymerase chain reaction (qPCR) analysis of ZNF471 mRNA levels in the indicated cell lines. (**C**) Top: western blot validation of ZNF471 overexpression in PC9-OR cells. Bottom: Cell viability curves of PC9-OR cells stably transfected with vector control or ZNF471 overexpression plasmid (oeZNF471) treated with serial concentrations of osimertinib. (**D**) Top: western blot validation of ZNF471 knockdown in PC9 cells. Bottom: Cell viability curves of PC9 cells infected with mock control or ZNF471 short hairpin RNA (shZNF471 #1 and #2) upon osimertinib treatment. (**E**) Transwell migration and invasion assays of PC9-OR cells (**left**) and PC9 cells (**right**). Scale bar, 100 μm. (**F**) Sphere formation assays of PC9-OR cells (**top**) and PC9 cells (**bottom**). Scale bar, 100 μm. (**G**) Colony formation assays of PC9-OR cells (**left**) and PC9 cells (**right**) under osimertinib treatment (0 or 2 μM). Statistical significance is denoted by *p < 0.05.

### 3.4 β-Hydroxybutyrate Reactivates ZNF471 Expression in Association with Promoter Demethylation in Lung Adenocarcinoma

Having observed that ZNF471 loss contributes to an osimertinib-resistant phenotype, we next sought to identify a pharmacological strategy capable of restoring ZNF471 expression. Because promoter hypermethylation was implicated in ZNF471 silencing, we examined whether β-hydroxybutyrate (BHB), an epigenetically active metabolite, could modulate ZNF471 expression in lung adenocarcinoma cells.

In EGFR-mutant lung adenocarcinoma cell models, treatment with 5 mM BHB for 24 h increased ZNF471 mRNA levels in both PC9 and H1975 cells (Fig. 4A). In PC9-OR osimertinib-resistant cells, BHB also increased ZNF471 mRNA expression in a concentration-dependent manner (Fig. 4B). Consistently, transcriptome sequencing of BHB-treated SPCA-1 and A549 cells independently identified ZNF471 as an upregulated gene (Fig. 4C). At the protein level, BHB treatment increased ZNF471 expression in PC9-OR cells in a concentration-dependent manner after 48 h (Fig. 4D). Time-course analysis further showed that ZNF471 upregulation induced by 5 mM BHB was detectable as early as 12 h and persisted through 72 h (Fig. 4E).

To explore whether this transcriptional reactivation was associated with promoter methylation changes, methylation-specific PCR was performed. BHB treatment increased the unmethylated ZNF471 promoter signal and/or reduced the methylated promoter signal in lung adenocarcinoma cell models, including SPCA-1, A549, PC9, and PC9-OR cells (Fig. 4F). Together, these findings suggest that BHB may reactivate ZNF471 expression, at least in part, in association with reduced promoter methylation. To explore a potential mechanism underlying BHB-associated reactivation of ZNF471, we analyzed an LC–MS/MS-based proteomic dataset from H1975 and A549 LUAD cells treated with 5 mM BHB for 24 h. DNMT3A, a *de novo* DNA methyltransferase, was reduced after BHB treatment in both cell lines compared with vehicle-treated controls (Supplementary Table S3). This finding provides exploratory evidence that decreased DNMT3A abundance may contribute to the attenuation of methylation-associated ZNF471 repression. Nevertheless, these data do not prove promoter-specific demethylation of ZNF471 and do not exclude additional epigenetic mechanisms, such as DNMT1/DNMT3B alteration, TET-mediated hydroxymethylation, histone acetylation, or histone β-hydroxybutyrylation. Further mechanistic validation will be required.

**Figure 4:**
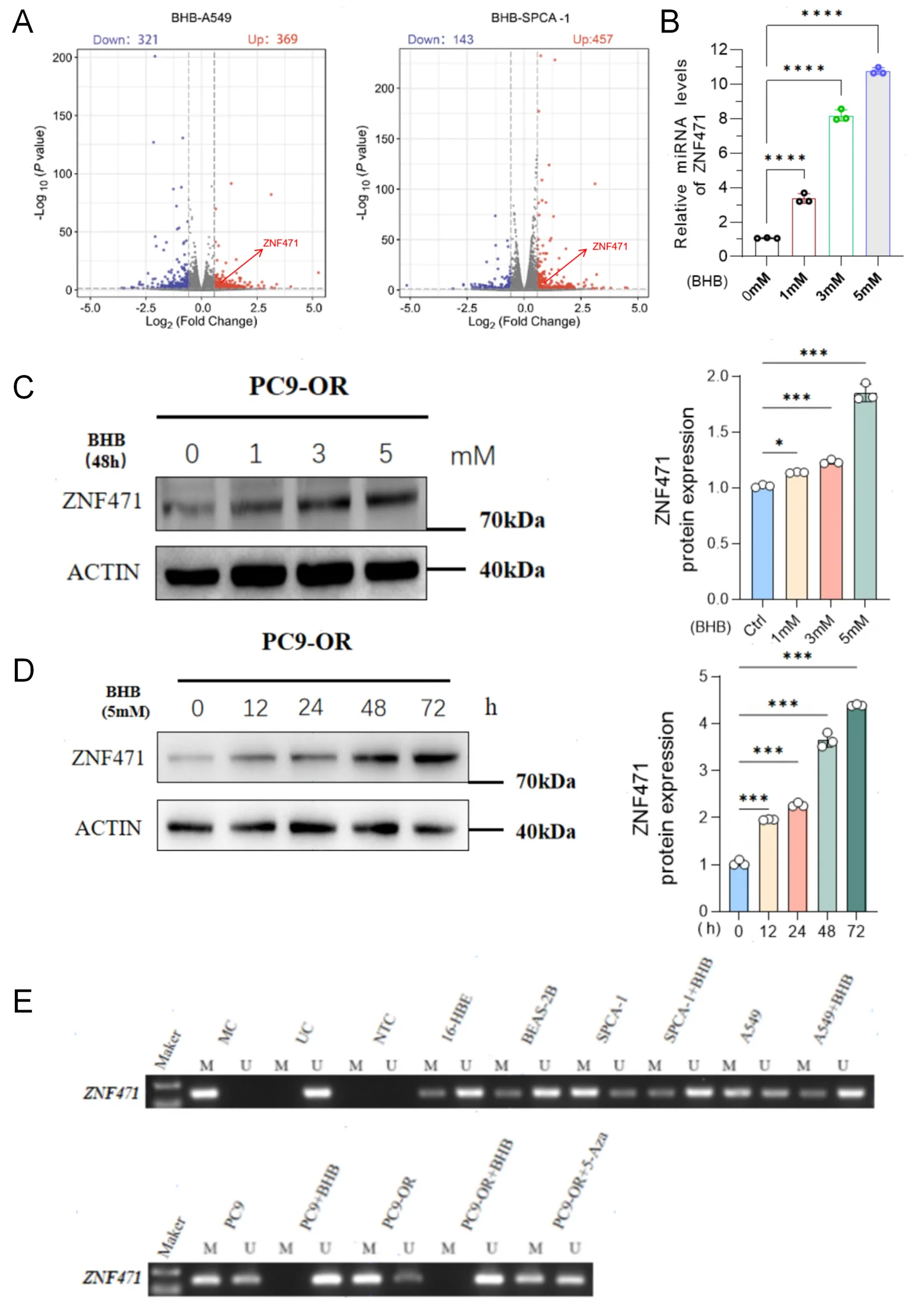
β-hydroxybutyrate increases ZNF471 expression and is associated with reduced promoter methylation signals in lung adenocarcinoma cells. (**A**) Quantitative polymerase chain reaction (qPCR) analysis of ZNF471 mRNA levels in PC9 and H1975 cells treated with 0 or 5 mM β-hydroxybutyrate (BHB) for 24 h. (**B**) qPCR analysis of ZNF471 mRNA levels in PC9-OR cells treated with increasing concentrations of BHB (0, 1, 3, and 5 mM). (**C**) RNA sequencing volcano plots comparing BHB-treated and control SPCA-1 and A549 cells. ZNF471 is highlighted as an upregulated gene. (**D**) Western blot analysis of ZNF471 protein expression in PC9-OR cells treated with increasing concentrations of BHB (0, 1, 3, and 5 mM) for 48 h. β-actin was used as the loading control. (**E**) Western blot analysis of ZNF471 protein expression in PC9-OR cells treated with 5 mM BHB for the indicated time points: 0, 12, 24, 48, and 72 h. β-actin was used as the loading control. (**F**) Methylation-specific PCR analysis of the ZNF471 promoter in the indicated lung epithelial and lung adenocarcinoma cell lines following BHB treatment. M, methylated PCR product; U, unmethylated PCR product; MC, methylated control; UC, unmethylated control; NTC, no-template control. Data are presented as mean ± s.d. from three independent biological replicates. Statistical significance was determined using an unpaired two-tailed Student’s *t*-test for two-group comparisons and one-way ANOVA followed by Dunnett’s multiple-comparison test for multiple-group comparisons. *p < 0.05, ***p < 0.001, ****p < 0.0001.

### 3.5 ZNF471-Mediated Attenuation of Osimertinib Resistance Is Accompanied by Reduced PI3K/AKT/mTOR Pathway Activity

Having established that BHB reactivates ZNF471 expression in association with changes in promoter methylation, we next examined the downstream signaling changes associated with ZNF471 restoration. Gene set enrichment analysis identified a significant negative enrichment of the HALLMARK_PI3K_AKT_MTOR_SIGNALING pathway in ZNF471-high LUAD samples (Fig. 5A). This finding was independently validated in the TCGA-LUAD cohort, where the ZNF471-high group exhibited markedly lower PI3K/AKT/mTOR pathway activity compared with the ZNF471-low group (Fig. 5B).

To investigate the downstream signaling events associated with ZNF471-mediated osimertinib resensitization, we examined key components of the PI3K/AKT/mTOR pathway by Western blotting. Restoration of ZNF471 expression in osimertinib-resistant LUAD cells reduced the phosphorylation levels of PI3K, AKT, and the mTOR downstream effector 4E-BP1, as reflected by decreased p-PI3K/PI3K, p-AKT/AKT, and p-4E-BP1/4E-BP1 ratios. In contrast, shRNA-mediated depletion of ZNF471 in parental LUAD cells increased phosphorylation of these signaling components (Fig. 5C,D).

Complete densitometric quantification of the Western blot results is provided in Supplementary Fig. S2. The quantified ratios include ZNF471/β-actin, AKT/β-actin, p-AKT/AKT, PI3K/β-actin, p-PI3K/PI3K, 4E-BP1/β-actin, and p-4E-BP1/4E-BP1. Statistical analysis showed that PI3K protein abundance was increased in the ZNF471-knockdown group. Together with the altered phosphorylation levels of PI3K and downstream effectorsthese findings support an association between ZNF471 expression and reduced PI3K/AKT/mTOR downstream signaling. However, pathway-rescue experiments are required to determine whether this signaling pathway is necessary for ZNF471-mediated reversal of osimertinib resistance.

**Figure 5:**
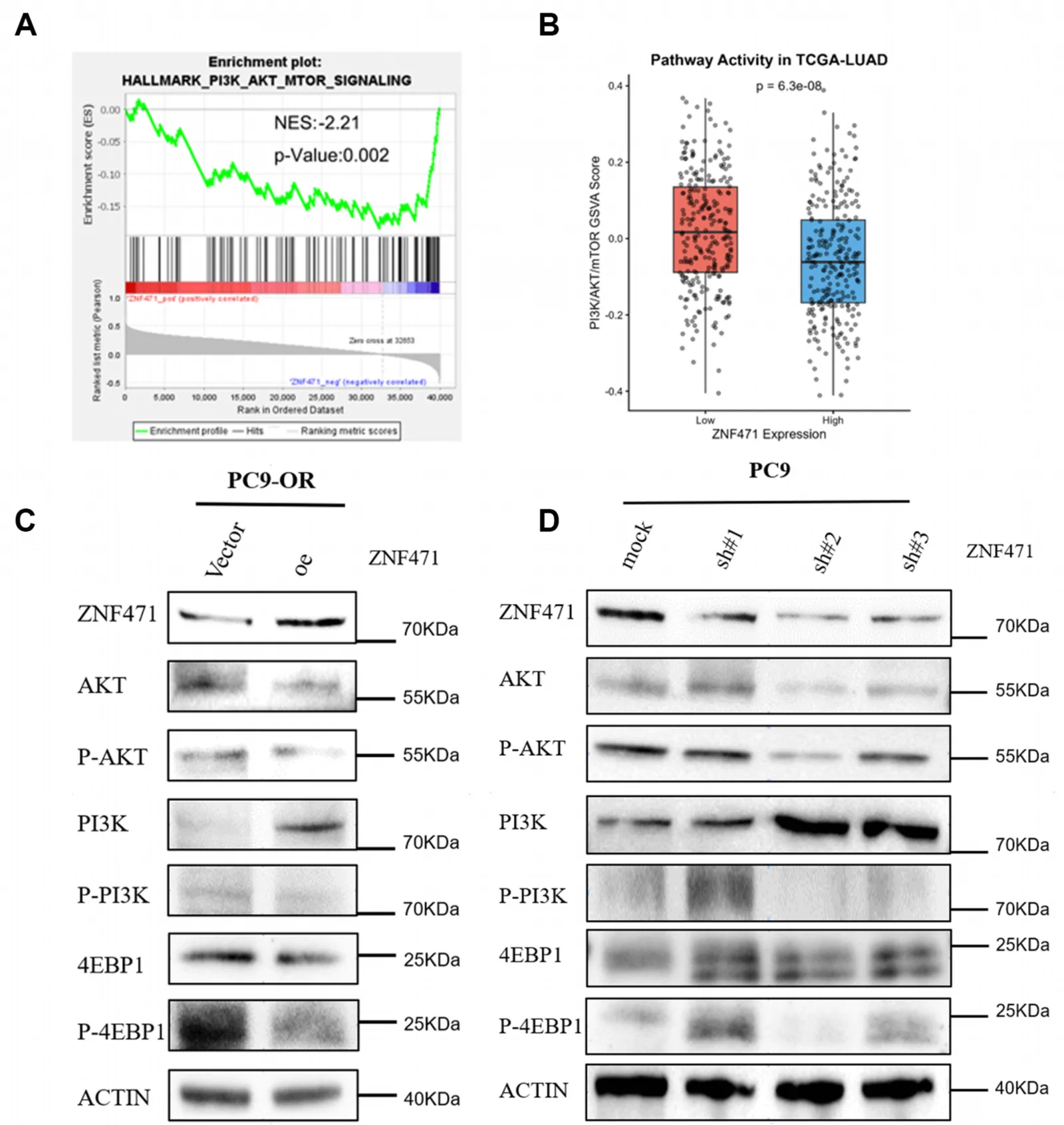
ZNF471 expression is inversely associated with PI3K/AKT/mTOR-related downstream signaling in LUAD. (**A**) Gene set enrichment analysis (GSEA) enrichment plot showing negative correlation between ZNF471 expression and HALLMARK_PI3K_AKT_MTOR_SIGNALING pathway in LUAD. (**B**) Gene set variation analysis (GSVA) validation in TCGA-LUAD cohort, demonstrating significantly lower PI3K/AKT/mTOR pathway activity in the ZNF471-high group compared with the ZNF471-low group. (**C**) Western blot analysis of ZNF471 and PI3K/AKT/mTOR-related pathway proteins in PC9-OR cells stably transfected with vector control or ZNF471 overexpression plasmid. (**D**) Western blot analysis of the indicated proteins in parental PC9 cells infected with mock control or shRNAs targeting ZNF471. β-actin served as the loading control. Densitometric quantification is provided in Supplementary Fig. S2.

### 3.6 β-Hydroxybutyrate Synergizes with Osimertinib to Overcome Drug Resistance in Lung Adenocarcinoma

The mechanistic link between ZNF471 restoration and PI3K/AKT/mTOR suppression prompted us to evaluate whether BHB, as a ZNF471-inducing agent, could enhance osimertinib efficacy in resistant cells. Notably, BHB alone exhibited minimal cytotoxicity toward PC9-OR and H1975-OR cells within the 0–5 mM range, whereas its combination with osimertinib markedly reduced cell viability in a concentration-dependent manner (Fig. 6A,B). Fa-CI analysis confirmed synergistic interaction between BHB and osimertinib in both resistant cell lines, with combination indices falling below the synergism threshold (Fig. 6C,D; Supplementary Tables S4 and S5). Beyond proliferation, BHB treatment significantly suppressed the migratory capacity of PC9-OR cells, as evidenced by reduced wound healing rates (Fig. 6E). These findings demonstrate that BHB sensitizes resistant cells to osimertinib through synergistic cytotoxicity and anti-migratory effects, independent of direct tumoricidal activity.

**Figure 6:**
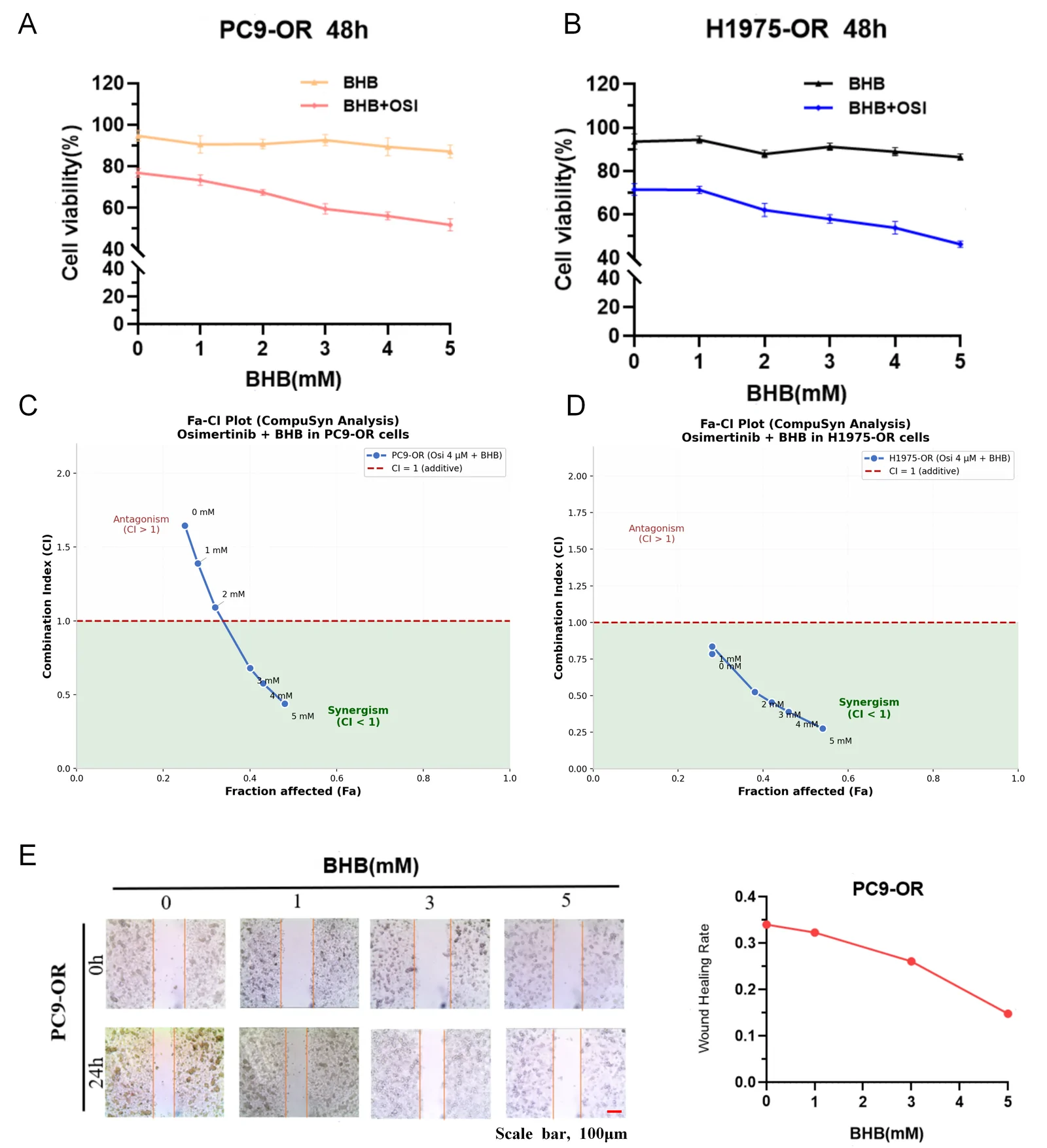
β-hydroxybutyrate (BHB) synergizes with osimertinib to inhibit osimertinib-resistant lung adenocarcinoma cells. (**A**,**B**) Cell viability of PC9-OR (**A**) and H1975-OR (**B**) cells treated with BHB alone (0–5 mM) or in combination with osimertinib (OSI) for 48 h. (**C**,**D**) Fa-CI plot (CompuSyn analysis) of osimertinib combined with BHB in PC9-OR (**C**) and H1975-OR (**D**) cells. Fa, fraction affected; CI, combination index; CI < 1 indicates synergism. (**E**) Wound healing assay of PC9-OR cells treated with BHB (0, 1, 3, and 5 mM) for 0 and 24 h, with quantification of wound healing rate. Scale bar, 100 μm.

### 3.7 BHB Treatment or ZNF471 Overexpression Attenuates PC9-OR Xenograft Growth

Encouraged by the synergistic efficacy of BHB and osimertinib observed *in vitro*, we extended our investigation to evaluate the therapeutic potential of ZNF471 restoration in an osimertinib-resistant xenograft model. In the pharmacological intervention arm, BHB treatment significantly suppressed PC9-OR tumor growth compared with vehicle control, without appreciable effects on mouse body weight (Fig. 7A,B, left). In the genetic intervention arm, ZNF471 overexpression similarly attenuated tumor volume and weight relative to vector control (Fig. 7A,B, right; Fig. 7C). Histological examination revealed that both interventions elevated intratumoral ZNF471 protein levels and reduced Ki67 proliferation index, indicative of diminished tumor aggressiveness (Fig. 7D). These *in vivo* findings are consistent with the *in vitro* observations and support the notion that BHB treatment or genetic restoration of ZNF471 can attenuate the growth of osimertinib-resistant LUAD xenografts.

**Figure 7:**
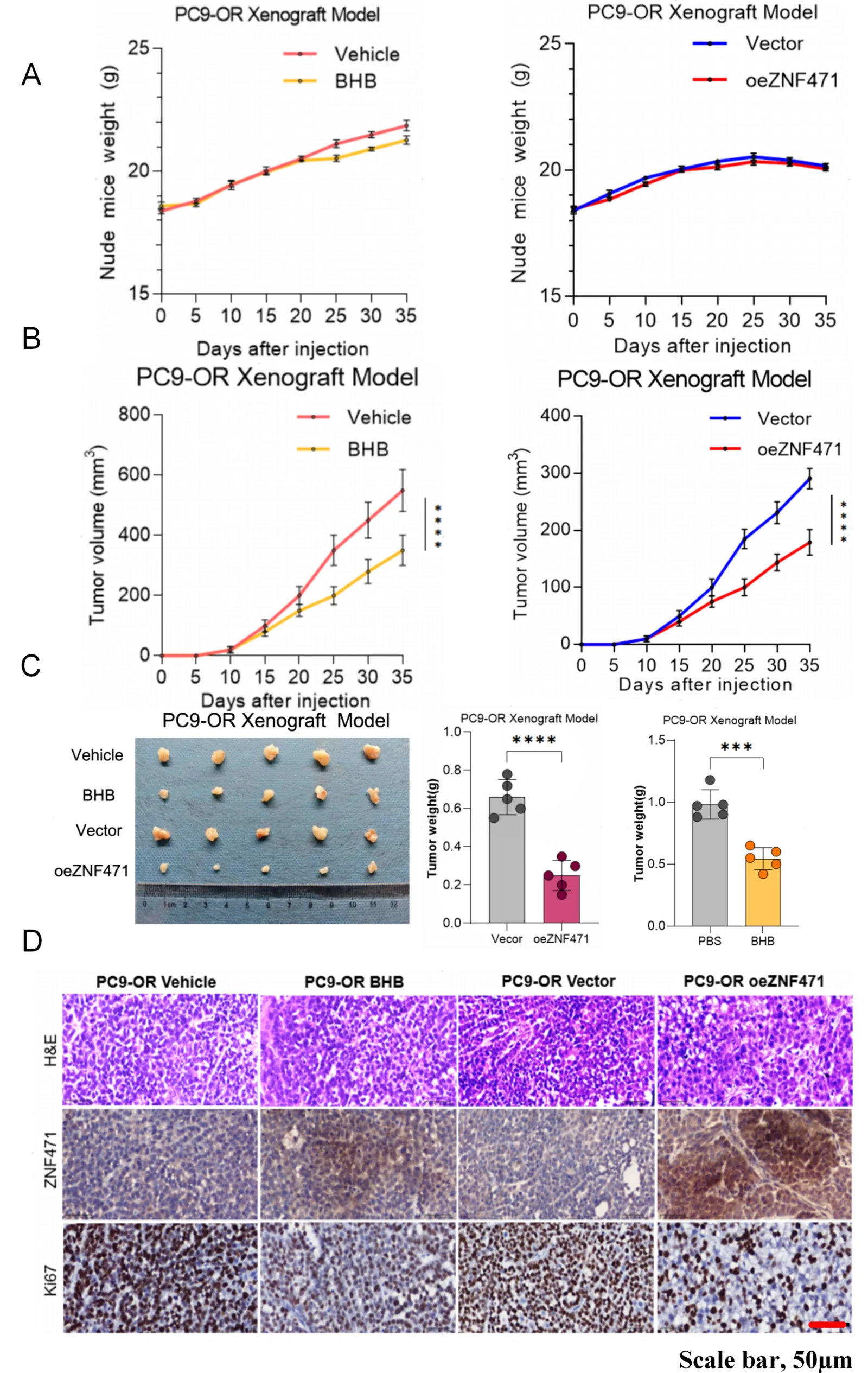
ZNF471 restoration suppresses osimertinib-resistant lung adenocarcinoma growth *in vivo*. (**A**,**B**) Nude mice bearing PC9-OR xenografts were treated with vehicle (PBS) or BHB. Body weight (**A**, **left**) and tumor volume (**B**, **left**) were monitored during treatment. Parallel experiment using PC9-OR xenografts stably transfected with vector control or ZNF471 overexpression plasmid (oeZNF471). Body weight (**A**, **right**) and tumor volume (**B**, **right**). (**C**) Representative tumor images at endpoint and tumor weight quantification for BHB-treated (**left**) and oeZNF471 (**right**) groups, respectively. (**D**) Hematoxylin and eosin (H&E) staining and immunohistochemical analysis of ZNF471 and Ki67 in tumor tissues from all four groups. Scale bar, 50 μm. ***p < 0.001, ****p < 0.0001.

## 4 Discussion

Disease progression after osimertinib should be interpreted not only as the acquisition of discrete genomic alterations but also as the outcome of therapy-driven tumor evolution, in which residual cancer cell populations adapt to drug pressure through dynamic transcriptional and phenotypic remodeling. Single-cell analyses of lung cancers exposed to targeted therapy have shown that drug-tolerant residual cells can pre-exist or emerge early during treatment and may later provide a cellular reservoir for clinically overt resistance [28,29]. This concept is consistent with broader evidence that non-genetic persister states, lineage plasticity, and reversible cell-state transitions substantially contribute to resistance to targeted anticancer therapies [29,30,31]. Because these adaptive states are not always captured by routine mutation-based profiling, resistance mechanisms mediated by chromatin remodeling, DNA methylation, and transcriptional reprogramming remain clinically important but incompletely defined [9]. In this context, our study supports ZNF471 promoter hypermethylation and transcriptional silencing as a functionally relevant epigenetic alteration associated with osimertinib resistance in lung adenocarcinoma, thereby linking a potentially reversible regulatory change to a clinically relevant resistant phenotype.

A major finding of this work is that ZNF471 is downregulated in lung adenocarcinoma and that reduced ZNF471 expression is associated with more aggressive clinicopathological features and unfavorable prognosis. Importantly, our methylation analyses indicate that ZNF471 repression is at least partly mediated by promoter CpG island hypermethylation, supporting an epigenetic silencing model rather than a nonspecific expression change. DNA methylation-mediated tumor suppressor silencing is a well-established mechanism by which cancer cells acquire malignant plasticity, adaptive survival capacity, and therapeutic resistance [9,32,33]. Therefore, the observed inverse relationship between ZNF471 promoter methylation and ZNF471 expression provides a biologically plausible explanation for its loss in lung adenocarcinoma.

Functionally, ZNF471 behaved as a suppressor of osimertinib-resistant phenotypes in our experimental models. Restoration of ZNF471 reduced osimertinib tolerance, impaired colony formation, migration, invasion, and stemness-associated properties in resistant cells, whereas ZNF471 depletion promoted resistance-associated phenotypes in sensitive cells. These reciprocal gain- and loss-of-function results suggest that ZNF471 is not only associated with clinical outcome but may also functionally contribute to the regulation of osimertinib-resistant phenotypes. This distinction is important because clinically useful resistance markers should ideally indicate not only disease behavior but also a modifiable therapeutic vulnerability [6,8].

Our reciprocal genetic manipulation experiments provide a plausible mechanistic explanation for the functional role of ZNF471 in osimertinib-resistant LUAD cells. Restoration of ZNF471 in PC9-OR cells attenuated the phosphorylation of PI3K, AKT, and the mTOR downstream effector 4E-BP1, whereas ZNF471 depletion in parental PC9 cells produced the opposite pattern. These findings are consistent with the possibility that ZNF471 restrains pro-survival PI3K/AKT/mTOR-related signaling, thereby contributing to reduced malignant phenotypes and improved osimertinib responsiveness. Nevertheless, the current data do not establish a fully linear or exclusive ZNF471–PI3K/AKT/mTOR signaling cascade. Additional pathway-rescue experiments, such as AKT activation using SC79 in ZNF471-restored cells or PI3K inhibition using LY294002 in ZNF471-depleted cells, will be required to confirm the causal dependency of these phenotypes on PI3K/AKT/mTOR signaling.

An important implication of this study is that methylation-associated silencing of a tumor suppressor may contribute to an experimentally modifiable osimertinib-resistant phenotype. While many studies have focused on stable genomic alterations as drivers of resistance, increasing evidence indicates that reversible epigenetic and transcriptional states can also shape therapeutic escape [6,8,9,34,35]. Unlike irreversible genetic events, epigenetic repression may be pharmacologically or metabolically reversible, creating a potential opportunity for therapeutic re-sensitization [36]. Thus, ZNF471 methylation may provide a molecular explanation for a subset of osimertinib-resistant LUAD and may serve as a candidate biomarker for identifying tumors potentially vulnerable to epigenetic-metabolic intervention.

Another important finding of this study is that β-hydroxybutyrate (BHB) increased ZNF471 expression and enhanced osimertinib sensitivity in resistant lung adenocarcinoma cells. BHB is increasingly recognized not only as an energy substrate but also as a signaling metabolite capable of modulating chromatin states and transcriptional programs through mechanisms such as histone deacetylase inhibition and lysine β-hydroxybutyrylation [24,25,37,38]. In our experiments, BHB induced ZNF471 expression in a concentration- and time-dependent manner and was associated with altered methylation-specific PCR patterns at the ZNF471 promoter. These findings suggest that BHB may act as a metabolic–epigenetic modulator that facilitates the reactivation of a silenced tumor suppressor associated with osimertinib resistance. However, because BHB can influence multiple epigenetic and metabolic processes, the specific enzymatic machinery responsible for ZNF471 promoter demethylation remains to be clarified.

The preclinical relevance of BHB-associated ZNF471 reactivation was supported by cell-based assays and xenograft experiments. In resistant cells, BHB had limited single-agent cytotoxicity at the tested concentrations but synergistically reduced cell viability when combined with osimertinib. BHB also reduced wound closure as a single agent, suggesting an additional effect on migratory behavior. In xenografts, BHB treatment or ZNF471 overexpression attenuated PC9-OR tumor growth without obvious body-weight loss. Because the animal experiments did not include a BHB–osimertinib combination arm, these findings should be interpreted as proof-of-concept evidence for BHB- or ZNF471-associated tumor suppression rather than definitive *in vivo* validation of combination therapy. Compared with broad epigenetic drugs, an endogenous metabolite-based approach may offer theoretical tolerability advantages, although clinical pharmacokinetics, tumor exposure, optimal dosing, and safety remain unresolved [39].

From a clinical perspective, our findings raise several hypotheses that warrant further investigation. First, ZNF471 expression or promoter methylation status may help identify a subset of tumors in which epigenetic repression contributes to osimertinib resistance [40]. Second, pharmacologic or metabolic approaches that restore ZNF471 expression could be explored as potential sensitizing strategies in combination with osimertinib, particularly in tumors showing PI3K/AKT/mTOR-related pathway activation [16,41]. These possibilities require validation in clinically annotated resistant cohorts and patient-derived models. These possibilities are consistent with the broader view that overcoming targeted therapy resistance will require integration of genomic, epigenomic, and phenotypic biomarkers rather than reliance on mutation profiling alone [42,43].

Several limitations should be acknowledged. First, although BHB treatment was associated with altered methylation-specific PCR patterns at the ZNF471 promoter, MSP is a qualitative or semi-quantitative assay and does not precisely define CpG-level methylation changes. Quantitative methylation assays, such as bisulfite sequencing or pyrosequencing, will be needed to confirm promoter-specific demethylation. Second, the proteomic analysis provided only exploratory evidence that methylation-related enzymes may be affected by BHB treatment. The observed reduction in DNMT3A abundance was not independently validated by qPCR or Western blotting, and additional epigenetic mechanisms, including DNMT1/DNMT3B alteration, TET-mediated hydroxymethylation, histone acetylation, chromatin accessibility changes, and histone β-hydroxybutyrylation at the ZNF471 locus, remain possible [12,44]. Third, although PI3K/AKT/mTOR-related signaling changed consistently after ZNF471 manipulation, pathway-rescue experiments are required to determine whether this pathway is necessary and sufficient for ZNF471-mediated modulation of osimertinib sensitivity. Fourth, the *in vivo* experiments provided proof-of-concept evidence for BHB treatment or ZNF471 overexpression but did not include an osimertinib-combination arm; therefore, the therapeutic efficacy of BHB plus osimertinib *in vivo* remains to be established. Finally, future studies should incorporate larger clinically annotated cohorts, paired pre-treatment and post-progression specimens, patient-derived organoids, and patient-derived xenografts to validate the predictive and therapeutic value of the proposed BHB–ZNF471 regulatory model.

## 5 Conclusion

In conclusion, our findings support ZNF471 as a promoter hypermethylation-associated candidate tumor suppressor linked to osimertinib sensitivity in LUAD. BHB treatment increased ZNF471 expression and was associated with reduced methylation-related repression, with exploratory proteomic data suggesting possible involvement of altered DNA methylation machinery. Functional experiments showed that ZNF471 restoration attenuated malignant phenotypes and enhanced osimertinib sensitivity, accompanied by reduced PI3K/AKT/mTOR-related downstream signaling. Together, these findings support a working model in which BHB-associated ZNF471 reactivation may contribute to osimertinib resensitization in LUAD. Further validation using ZNF471 rescue experiments, quantitative methylation assays, chromatin analyses, pathway-rescue studies, and clinically defined osimertinib-resistant patient cohorts will be required.

## Data Availability

The data that support the findings of this study are available from the Corresponding Author, Chongmei Liu, upon reasonable request.

## Supplementary Materials

The supplementary material is available online at https://www.techscience.com/doi/10.32604/or.2026.087712/s1.

## Abbreviations

AKT	protein kinase B
ANOVA	analysis of variance
ATCC	American Type Culture Collection
BHB	β-hydroxybutyrate
CCK-8	Cell Counting Kit-8
cDNA	complementary DNA
CI	combination index
CpG	cytosine-phosphate-guanine dinucleotide
DNA	deoxyribonucleic acid
DNMT	DNA methyltransferase
DSS	disease-specific survival
EGFR	epidermal growth factor receptor
Fa	fraction affected
FBS	fetal bovine serum
GDC	Genomic Data Commons
gDNA	genomic DNA
GEO	Gene Expression Omnibus
GSEA	gene set enrichment analysis
GSVA	gene set variation analysis
H&E	hematoxylin and eosin
HR	hazard ratio
IC_50_	half maximal inhibitory concentration
IHC	immunohistochemistry
LUAD	lung adenocarcinoma
MSP	methylation-specific PCR
mTOR	mechanistic target of rapamycin
OS	overall survival
OSI	osimertinib
PBS	phosphate-buffered saline
PD	progressive disease
PI3K	phosphoinositide 3-kinase
PVDF	polyvinylidene fluoride
qPCR	quantitative polymerase chain reaction
RNA	ribonucleic acid
RNA-seq	RNA sequencing
RPMI	Roswell Park Memorial Institute
RT-qPCR	reverse transcription quantitative PCR
s.d.	standard deviation
SD	stable disease
SDS-PAGE	sodium dodecyl sulfate polyacrylamide gel electrophoresis
shRNA	short hairpin RNA
STR	short tandem repeat
TCGA	The Cancer Genome Atlas
TET	ten-eleven translocation
TKI	tyrosine kinase inhibitor
TPM	transcripts per million
ZNF471	zinc finger protein 471
4E-BP1	eukaryotic translation initiation factor 4E-binding protein 1
