# Supplementary material for: Epigenetic Silencing of ZNF471 Contributes to Osimertinib Resistance in Lung Adenocarcinoma: β-Hydroxybutyrate Associates with ZNF471 Re-Expression and Reduced Resistance-Associated Phenotypes
Source: Oncol Res. 2026 Oct 28;34(11):24. doi: 10.32604/or.2026.087712 (PMC13732338; doi:10.32604/or.2026.087712)
Supplement: Supplementary file 1 [file OncolRes-34-87712-s001.zip › TSP_OR_87712-s001.docx]

**Supplementary information**

**Supplementary Figure S1:** Quantification of ZNF471-mediated effects on colony formation, migration/invasion, and tumorsphere formation in LUAD cells. (A) Transwell migration and invasion assays were quantified as the number of stained cells per field. (B) Sphere formation was quantified as the mean tumorsphere diameter (μm). (C) Colony formation was quantified as the number of colonies within the standard size range in PC9-OR/vector and PC9-OR/oeZNF471 cells, and in PC9/mock, PC9/shZNF471#1, and PC9/shZNF471#2 cells, under low- or high-density seeding conditions with or without 2 μM osimertinib. Data are presented as mean ± SD. Statistical significance is indicated as ns, not significant; **P < 0.01; ***P < 0.001; ****P < 0.0001.

**Supplementary Figure S2:** ZNF471 knockdown partially rescued clonogenic survival of PC9 cells under osimertinib treatment. Consistently, densitometric analysis of Western blots confirmed efficient modulation of ZNF471 expression and indicated that ZNF471 overexpression was associated with reduced phosphorylation ratios of AKT, PI3K, and 4EBP1, while ZNF471 depletion induced shRNA-dependent alterations in the PI3K/AKT/4EBP1 signaling axis. ns, not statistically significant, ***P < 0.001, ****P < 0.0001.

**Supplementary Table S1. Sequences of primers**

| **Gene** | **Primer type** | **Sequence (5′–3′)** | **Product size (bp)** |
| --- | --- | --- | --- |
| **ZNF471** | **Forward primer** | **GTTGGAGAACTATCAGAGCCTGG** |  |
|  | **Reverse primer** | **GGCTTCTTGTCATCTCACTCGTC** |  |
| **ZNF471 M** | **Forward primer** | **TTTAGGAATTAGTAATTAGGGACGT** | **168bp** |
|  | **Reverse primer** | **AAACCTTAAAACAAAACGAACGAT** |  |
| **ZNF471 U** | **Forward primer** | **TTTAGGAATTAGTAATTAGGGATGT** | **169bp** |
|  | **Reverse primer** | **AAAACCTTAAAACAAAACAAACAAT** |  |
| ***β*-actin** | **Forward primer** | **CACCATTGGCAATGAGCGGTTC** |  |
|  | **Reverse primer** | **AGGTCTTTGCGGATGTCCACGT** |  |

**Supplementary Table S2. RNAi sequences**

| **Gene** | **Sense sequence (5’ to 3’)** |
| --- | --- |
| **Negative Control** | **UUCUCCGAACGUGUCACGUTT** |
| **shZNF471-1** | **ACATCTTTTTTATTCGTCACTGGAGGAGGATTATCATACTGGAGGAGA** |
| **shZNF471-2** | **CTGCTCAGAAGCGTTTATACAGGAGTATGATGTTGGAGAAC** |
| **shZNF471-3** | **AGCCAAACTTCCAATCTTACTCAACATCAAAGAATTCATAC** |
| **shNC** | **Scrambled** |

**Supplementary Table S3. Proteomic data for methylation-related enzymes**

| **Protein accession** | **Gene name** | **H1975_trea/H1975_ctrl Ratio** | **A549_trea/A549_ctrl Ratio** | **Direction (BHB vs Vehicle)** | **Intensity H1975_ctrl** | **Intensity H1975_trea** | **Intensity A549_ctrl** | **Intensity A549_trea** |
| --- | --- | --- | --- | --- | --- | --- | --- | --- |
| Q9Y6K1 | DNMT3A | 0.649534403 | 0.928282886 | Decreased | 58.58865 | 38.05534 | 61.35287 | 56.95282 |

**Supplementary Table S4. Effect and CI of BHB and osimertinib combined treatment in PC9-OR**

| Dose BHB (mM) | Dose osimertinib | Effect | CI |
| --- | --- | --- | --- |
| 0 | 4μM | 0.25 | 1.646 |
| 1 | 4μM | 0.28 | 1.389 |
| 2 | 4μM | 0.32 | 1.092 |
| 3 | 4μM | 0.40 | 0.680 |
| 4 | 4μM | 0.43 | 0.578 |
| 5 | 4μM | 0.48 | 0.440 |

**Supplementary Table S5. Effect and CI of BHB and osimertinib combined treatment in H1975-OR**

| Dose BHB (mM) | Dose osimertinib | Effect | CI |
| --- | --- | --- | --- |
| 0 | 4μM | 0.28 | 0.758 |
| 1 | 4μM | 0.28 | 0.836 |
| 2 | 4μM | 0.38 | 0.525 |
| 3 | 4μM | 0.42 | 0.453 |
| 4 | 4μM | 0.46 | 0.389 |
| 5 | 4μM | 0.54 | 0.276 |

Footnotes:

•	CI: Combination index. Fa: Fraction affected = 1 − (cell viability / 100). The fixed concentration of osimertinib was 4 μM. Synergism: CI < 1; Additive effect: CI = 1; Antagonism: CI > 1.

•	The combination index (CI) was calculated using CompuSyn software based on the Chou-Talalay median-effect principle: CI = (D)₁/(Dx)₁ + (D)₂/(Dx)₂, where Dx = Dm × (Fa/Fu)^(1/m) and Fu = 1 − Fa.
